# Patient Perspectives on Short-Course Pharmacotherapy: Barriers and Facilitators to Medication Adherence

**DOI:** 10.1177/2374373519882230

**Published:** 2019-10-23

**Authors:** LeeAnne B Sherwin, Diana Ross, Michelle Matteson-Kome, Matthew Bechtold, Chelsea Deroche, Bonnie Wakefield

**Affiliations:** 1 Sinclair School of Nursing, 2628 University of Missouri, Columbia, MO, USA; 2 Department of Gastroenterology, University of Missouri Healthcare, 2628 University of Missouri, Columbia, MO, USA

**Keywords:** medication adherence, irritable bowel syndrome, patient perspectives/narratives, short-course pharmacotherapy

## Abstract

**Background:**

Medication nonadherence is a public health issue that contributes to poor health outcomes and health-care costs. Factors influencing long-term medication adherence are known; however, little is known about short-course medication adherence.

**Objective:**

This study examined patient perspectives on adherence and factors that influence adherence to short-course pharmacotherapy in diarrhea-predominant irritable bowel syndrome.

**Method:**

Twenty-seven participants were interviewed to identify their perceptions of barriers and facilitators to thrice-daily, 14-day rifaximin.

**Results:**

Participants were primarily female (89%), aged 18 to 65 years. Sixty-eight percent of interviewees were identified as “low-adherers,” meaning the percentage of days with correct daily dosing of rifaximin was <80%. The final coding framework identified social/economic-related (family support and medication expense), system-related (relationship with provider and medication knowledge), condition-related (symptom severity), therapy-related (inconvenient dosing), and patient-related (forgetfulness and busyness of daily life) factors that influenced adherence.

**Conclusion:**

The resulting patient perspectives highlight a diverse set of factors that influence short-course adherence and the need for tailored interventions that address these various factors resulting in enhanced patient outcomes.

## Introduction

Medication adherence refers to the level of a patient’s participation in taking their medications as prescribed (1–3). Nonadherence is a global public health issue. In the United States, it is estimated that 1 in 5 new prescriptions written are not filled and of those filled, half are not taken as directed (4,5). Nonadherence can occur at any of stage (initiation, implementation, or persistence) within the adherence process (2). Five dimensions have been identified as key factors that influence medication adherence (5). These factors include social and economic, healthcare system, condition-related, therapy-related, and patient-related. Within each of these, numerous factors are associated with nonadherence. These dimensions and the associated factors have been investigated in studies of long-term pharmacotherapy for chronic illnesses (5–8); however, few studies have investigated these dimensions in short-course pharmacotherapy in the treatment of chronic illnesses as opposed to treatment of acute infections (9,10).

Diarrhea-predominant irritable bowel syndrome (IBS-D), a common chronic gastrointestinal disorder characterized by abdominal pain and diarrhea, is often treated with an antibiotic to relieve overall IBS-D symptoms (11,12). The course of treatment is short term but considered complex with a thrice-daily intake for 14 days. The complexity of this treatment lends itself to potential medication nonadherence (5).

To effectively challenge nonadherence and improve outcomes, researchers and clinicians need an understanding of the barriers and facilitators of medication adherence and interventions that would be effective and feasible for enhancing adherence (5). Successful interventions for medication adherence enhancement are crucial to improving chronic disease outcomes and decreasing health-care costs. To our knowledge, there are no studies that have focused on short-course pharmacotherapy adherence in IBS-D. Our study sought to broaden the understanding of the patients’ perspective regarding short-course medication adherence and gain feedback on potential intervention strategies.

## Methods

### Study Design and Participants

Participants were recruited from the Psychosocial Determinants of Rifaximin Adherence for the Treatment of Irritable Bowel Syndrome (PDRATx-IBS) parent study. This longitudinal study collected substantial quantitative data on rifaximin adherence. Objective adherence was assessed with the Medication Event Monitoring System cap (13). PDRATx-IBS study design, recruitment, and findings have been previously described elsewhere (14). In brief, PDRATx-IBS participants included 73 adult patients receiving 14 days of thrice-daily rifaximin treatment for IBS-D. Both the parent study and the current study were approved by the University of Missouri Institutional Review Board. The PDRATx-IBS participants were eligible to participate in the qualitative study if they indicated that they were interested in being contacted at the completion of their 14-day prescription for a telephone interview inquiring about their perceptions regarding barriers and facilitators of rifaximin adherence and provide feedback regarding proposed intervention development. Recruitment occurred from January to September 2018.

### Data Collection and Analysis

Individual telephone calls were completed to interview participants regarding barriers and facilitators to rifaximin adherence. Participants were recruited to participate in telephone interviews until saturation of the data was achieved. We used semistructured, open-ended questions to guide the interviews and allowed the participants to describe their thoughts regarding barriers and facilitators to rifaximin adherence. Follow-up questions for clarification were asked based on the information provided by the participant. There were 2 interviewers: a PhD-prepared nurse researcher with expertise in gastroenterology and research (L.B.S.) and a PhD student experienced in qualitative interviews (D.R.). Upon completion of the telephone interview, participants were offered US$25 check in appreciation for their time. Interviews were audiotaped and transcribed verbatim and then analyzed using inductive content analysis. The deidentified transcripts were manually coded (D.R.), categories were identified, and then grouped for analysis. The primary (D.R.) and secondary (L.B.S.) coders reviewed and reconciled differences. Data management software was used (15). Data were reviewed by secondary coder (L.B.S.) to ensure code accuracy and to identify important subthemes. A coding memo that summarized findings and example quotations was developed and reviewed by the research team for face validity, breadth of analysis, and themes to be explored.

## Results

Twenty-seven participants completed the interviews at which time saturation was obtained. Table 1 reports participant characteristics. The mean interview time was 17 minutes. Twenty-six participants were interviewed via phone; per participant request, 1 interview was completed in private at the participating health clinic. Dimension and factor categories of the identified barriers and facilitators to short-course medication adherence with illustrative quotes are provided in Tables 2 and 3.

**Table 1.** Characteristics of Participants.^a^

Variable	n
Gender	
Female	24
Male	2
Nongender conforming	1
Age	
18-29	6
30-39	5
40-49	4
50-65	12
Race	
Caucasian	27
Marital status	
Married/living with partner	13
Single/never married	6
Divorced	6
Widowed	2
Time since diagnosis	
<12 months	7
12 months to 5 years	6
5 years to 10 years	6
>10 years	8
Participants who took daily medications	15
Adherence level	
Low adherers	15
High adherers	6
Did not initiate medication	6

^a^ “Low-adherers” is defined as a participant whose percent of days with correct daily dosing of rifaximin was <80% during the 14-day prescription. Alternately, “high-adherers” achieved 80% or greater adherence to the prescribed daily dosing.

**Table 2.** Barriers to Short-Course Medication Adherence Illustrative Quotes.

Dimensions	Factors	Illustrative Quote
Social and economic-related	Restricted formulary	“If the insurance ain’t going to cover it, I am not going to pay for it! I just cannot afford to pay for it all out of my wallet”
	Family/friend support	“My husband would turn off my alarm and forget to tell me”
Healthcare system-related	Patient–provider relationship	“The doctor didn’t tell me anything about it [rifaximin] so I figured why should I take it, I don’t know if it’s gonna work, so why bother”
Condition-related	Symptom severity	“I think I started to forget to take it [rifaximin] because I felt so good. I kept forgetting to take it…I wasn’t focused on taking it like in the beginning.”
Patient-related	Forgetfulness	“I kept forgetting, I don’t take pills always…I would forget to take any of the doses. It didn’t matter. I would remember but I would get busy especially at work and forget”
Therapy-related	Complexity of medication regimen	“The biggest challenge was just remembering to take it. I don’t usually take medicines so now taking it 3 times a day was hard to get into the groove of things.”
		“I thought I could do anything for 14 days. I was good for a few days but it was so hard to get that middle dose”
		“Just keeping track sometimes, did I already take this at dinnertime?…keeping track of when I had taken it was a challenge, I would forget and wasn’t sure if I should take it or did I already take it.”
	Duration of therapy	“I couldn’t get into a routine, I mean I thought about it but it was so short [length of prescription].”

**Table 3.** Facilitators to Short-Course Medication Adherence Illustrative Quotes.

Dimensions	Factors	Illustrative Quote
Social-related	Family/friend support	“My mother texted me every day at lunch to remind me to take it [rifaximin]”
Healthcare system-related	Patient–provider relationship	“I have seen a lot of doctors for this. It was the first time that I had seen this doctor. When I saw her, she had all the information, all of that was in her hands before I got there, and she’d read it, all of it!…She came in knowing exactly what I had been dealing with, had a really good handle on it. So when she recommended the medication, I was so impressed I felt very comfortable so I thought she knows her stuff, I’ll take it and I did.”
	Shared decision-making	“The doc listed so many options to treat this, food, antibiotics, and other stuff, you name it, he listed them…So we talked pros and cons. I decided on the rifaximin. It was all me. I felt in control but he guided me to make a good decision for me…I was going to take it because I wanted to take it not because he told me to take it.”
Patient-related	Motivation and confidence	“I take other pills morning and evening so I was good, I just needed to remember the middle dose, and I think I was able to remember pretty good because I thought…I have to take the pill with lunch, I can remember to do that. I was really good but I know I missed one or two.”
	Perceived benefit of treatment	“I could see it [diarrhea frequency] start to change, it made it easier to take it [rifaximin].”
Condition-related	Symptom severity	“I saw a change so I kept on taking it like I should. I was basically encouraged there was something that works for me.”
Therapy-related	Perceived side effects	“I always have problems, with this no problem…so I was able to complete it”

### Barriers and Facilitators of Short-Course Pharmacotherapy

#### Social and economic-related factors

Participants from the PDRATx-IBS parent study who did not fill their prescriptions were asked, prior to withdrawal from the study, why they did not fill their prescription. All 6 participants reported that the cost of the medication due to no insurance coverage or lack of insurance formulary coverage was a barrier to filling the prescription. These participants decided not to pay out of pocket for their medication. Therefore, medication cost and lack of insurance formulary coverage, which was categorized under economic-related factors, were identified as a barrier to adherence in this subgroup. Although for some participants the out-of-pocket expense was a factor that encouraged them to take all of the medication, one participant equated the high cost of the medication as indicative of high quality and efficacy and thought it best to complete the medicine course.

#### Healthcare system related

There was an overlap between economic barrier related to out-of-pocket expense due to restricted formulary and healthcare system-related issue of restricted formulary. Although participants identified the cost of their medication as a barrier that was categorized within economic factors, it is important to mention that the cost for several participants was directly related to the restricted formulary within their insurance provider. The lack of formulary coverage contributed to 6 participants’ decision not to initiate their rifaximin prescription. Thus, dual categorization for economic reasons related to system constrictions.

Five of the 27 participants noted that a delay in filling the prescription was directly attributed to waiting for insurance authorization for prescription payment. Participants attributed the approval delay directly to their delay in the initiation of the medication. This delay resulted in rescheduled of their follow-up visits that resulted in scheduling with alternate provider contributing to care discontinuity.

Several participants mentioned that their interaction with their provider was a facilitator for initiating and continuing the medication. Preparedness of the provider and open communication and shared decision-making regarding potential treatments were identified as reasons for initiating their prescriptions. Additionally, it was noted that having a conversation about potential treatments with their provider was an important factor to initiating and completing their prescription.

Another system-related factor that several participants identified as a facilitator was the ease of obtaining the medication through the mail or through the same day prescription filling from the in-office pharmacy. Participants commented that not needing a separate pharmacy visit helped to enhance the time to initiation of their medication. One participant was noted in saying “It [rifaximin] arrived in my mailbox, so I just started it that day. It was great.”

#### Condition related

The majority of participants reported moderate-to-severe diarrhea symptoms prior to initiating their therapy. Several of them reported their diarrhea symptoms lessening after day 4 of rifaximin. They further went on to report that because they saw a reduction in the diarrhea severity, they decided to continue their medication.

#### Therapy related

Remembering to take their medication was repeatedly reported as a major barrier to adherence. The majority of participants stated that the complexity of the medication regimen contributed to their difficulty in remembering to take their mid-day and evening doses of rifaximin. Although several of the participants initially thought the 14-day therapy as “easy to manage,” at the end of therapy, several admitted experiencing challenges with establishing a routine and completing the rifaximin as prescribed.

#### Patient related

A few participants noted that although they set alarms on their phones, family members would silence the alarm and either forget to notify them or the family member would inform them, but then the participant would forget to take the medicine due to their busy schedules.

Several participants noted that they were unsure how frequently to take their medication. They reported this lack of confidence or lack of knowledge of when to take their medication was a barrier that contributed to inconsistent daily intake of the rifaximin. Alternately, one participant described how their confidence with their routine helped them to take their medication.

A number of participants shared that “being told” or reading about the benefits of the medication was helpful in continuing the course of treatment. Participants specifically identified a reduction in the frequency of bathroom visits as a major reason for completion of the rifaximin prescription.

### Patient-Identified Strategies to Enhance Adherence

Participants were asked about potential strategies that would be effective in overcoming their forgetfulness, establishing a routine was the number one reported strategy. Suggestions for using a pill reminder case, reminder sticky notes, and placing the prescription bottle near where they ate were all mentioned.

One strategy identified addressed the therapy-related dimension through packaging changes. Incorporating packaging changes were thought to minimize concerns regarding complexity and duration of the medication intake in addition to addressing potential language proficiency issues. Although language was not identified as a direct barrier in our study, one participant was noted as saying, “If the package had symbols…wouldn’t even have to read, you would know when to take the pills.” Additionally, 2 participants identified that they believed packaging changes have the potential to enhance adherence:

Apps and alarms are ok the time that it [rifaximin] is taken. I mean I wouldn’t want it for my other pills. I like the pill minder box for that. That [pill reminder box] helps me remember. But you know what, you know what would really help, how about one of those blister packs like what steroids come in, how about that design? Then you know that you took what dose you need to take. No counting, you see it right there, you know I took it or not. I think that would have helped me a lot.

The second said,

You know those blister packs, I think that would be easier for people to remember to take them [rifaximin] and also to see that they haven’t taken it because when you just keep it in a pill bottle you’re not sure. It’s like, okay, did I take it or not? If it’s in a blister pack you just know you’ve taken that one.

To gain feedback on potential strategies for interventions that could be designed to lessen the patient-related forgetfulness, participants were asked about the feasibility and acceptability of alarms, text reminders, or smartphone apps specifically designed as medication reminders. All but one participant had a smartphone. Several participants felt using an alarm had the potential to be helpful. One participant said: “I put a reminder in my phone. An alarm went off. I didn’t follow it exact but I got, you know, got as close as I could.” Whereas several other participants reported that hearing an alarm or receiving a text reminder 3 times a day would be “too much.” One reported:

I mean it [text reminder] might be good for some. For me probably but if I didn’t have the container with me or if I forgot to bring that container with me it’s not gonna do any good cause I’m not at home, cause I work. I’m a teacher and I could check it at noon, so it would help me with that midday dose I guess but I would need to have it with me and that bottle was too bulky to have.

Other strategies used to help remember included writing on a calendar, both electronic and paper, a dry erase board posted on the refrigerator, and sticky notes posted on purses and doors. These methods were used as reminders and to keep track of doses taken. One participant reported, “I eventually started writing down and crossing off the times so that I would remember.”

Participants were asked if inclusion of family/friends would have the potential to enhance their adherence, specifically participants were asked if having their family/friend(s) receive text notification of their medication intake as beneficial. Several participants identified their family/friend(s) as busy and unsure if they would participate; however, a greater proportion noted inclusion of family/friend(s) as having the potential to be helpful. Two participants reported that they received reminders from family member/friend. One participant thought receiving a reminder from their family/friend would make them feel less guilty if they missed a dose. They did not like the idea of a text reminder coming from their doctors’ office. One said:

It [text reminder] makes me feel sick. Well I know I am sick but this is what I live with all the time and I am not sick like a diabetic, I have diarrhea all the time, yeah it is disrupting but it’s not like I am sick so I guess I’d rather talk to my mom and have her remind me than the doctor.

## Discussion

This study explored patient perspectives about barriers and facilitators as well as identifying patient-centered strategies focused on enhancing short-course pharmacotherapy in adults who have IBS-D. Several factors were identified as influencers of adherence. These included social/economic-related, healthcare system-related, patient-related, condition-related, and therapy-related factors.

This study is the first to examine facilitators and barriers to short-course pharmacotherapy in IBS from the patient’s perspective. The results provide new information on patient perspectives regarding short-course pharmacotherapy for the treatment of a gastrointestinal chronic disease. Much of the previously published qualitative research focuses on long-term pharmacotherapy in chronic disease (16–18). The results of our study report similar barriers and facilitators to adherence that are reported for long-term pharmacotherapy.

Several factors were identified as influencers of short-course pharmacotherapy adherence. Complexity of the medication regimens is known to influence adherence to long-term medications (5). Similarly, participants identified having to take their rifaximin 3 times daily was challenging and contributed to their unintentional nonadherence. In the PDRATX-IBS parent study, 92% of participants were nonadherent to their daily dosing regimen; the midday dose was the most frequently missed dose. Participants acknowledged that the midday dose was the most difficult to remember. Nonadherence, whether through omission of a dose or delay in taking a dose, is the most common deviations reported in the literature (14,19). Forgetfulness was attributed as a barrier to taking the midday dose in this study. Decreasing the frequency of medications has been associated with better adherence (20). Simplifying the dosing regimen may be an important option for maximizing therapeutic success, thus resulting in decreased medical resource consumption. However, modifying the current treatment regimen is not a simple task. Additional research to explore and develop a treatment algorithm that is both simple and efficacious would be needed.

The second major factor identified by participants as negatively influencing their adherence was forgetfulness. Forgetfulness has been reported as the most common factor associated with medication nonadherence (5,21). Although the pharmacotherapy in this study was short, participants reported challenges with remembering to take their daily doses as directed. Unintentional nonadherence has been reported highest in those who are white, young to middle-aged, college educated, working, and report a high self-rated health (22). Our participants were primarily of this demographic. The literature reports the greatest challenges with adherence that occur in the first 6 months of therapy (23). Our study provides new information that challenges unintentional nonadherence to occur in short-course therapy as well. Interventions that focus on reminders and establishing a medication regimen have the potential for the greatest influence on minimizing forgetfulness.

The third major factor, which negatively influenced the initiation of rifaximin, was cost. Participants reporting high insurance copays and/or having to shoulder the full cost chose not to fill the prescription. Financial and cost constraints have been reported as preventing patients from adhering to long-term medication regimens (24). Discussing costs with the patient and utilizing pharmaceutical company-sponsored discount vouchers may provide the support patients need in order to fill their prescriptions.

### Strengths and Limitations

Advantages of focus groups compared to individual interviews has been debated over the years (25–28). Numerous strengths and weaknesses have been described for both. Our study utilized individual telephone interviews to decrease the burden for participant travel, scheduling convenience, and respondent anonymity/privacy. The resulting interview discussions provided rich data that would otherwise not have been available through the quantitative PDRATx-IBS parent study. Another potential limitation is that slightly more than 40% of participants were over the age of 50. Cognitive functioning was not evaluated in this study. It is possible that participants over 50 had undiagnosed mild cognitive impairment, thus contributing to the forgetfulness barrier reported. Evaluation of cognitive functioning in future adherence research should be considered. Additionally, participants were asked if they took a pharmaceutical treatment for depression and/or anxiety. Seventy-one percent reported taking medication for the depression, anxiety, or both; however, we are unaware of the daily dosing regimen for these treatments. Nor did we did inquire about other daily prescribed medications or nonprescription supplement intake frequencies. It is possible participants pharmaceutical intake influenced their adherence. Although participants reported pharmaceutical treatment for psychological distress, 70% were categorized as low adherers. In future studies, evaluating the role of polypharmacy on adherence may provide additional details regarding barriers/facilitators to adherence. The final limitation to this qualitative study was the minimal diversity of both sex and race, which contributes to the limited generalizability of the findings.

## Conclusion

Our study presents the patients’ perspectives of barriers and facilitators to short-course pharmacotherapy for the treatment of IBS-D in adults. In addition, participants also provided feedback regarding potential interventions directed at enhancing short-course medication adherence. Knowledge of the challenges experienced by patients, as reported through their voice, undergoing complex short-course pharmacotherapy provides the evidence needed to develop patient-centered interventions resulting in improve adherence. Developing and implementing interventions that incorporate these patient-centered strategies have the potential to enhance adherence and improve outcomes.
